# Supplementary material for: Effectiveness of aerobic exercise in the prevention and treatment of postpartum depression: Meta-analysis and network meta-analysis
Source: PLoS One. 2023 Nov 29;18(11):e0287650. doi: 10.1371/journal.pone.0287650 (PMC10686497; doi:10.1371/journal.pone.0287650)
Supplement: S2 Table — (DOCX) [file pone.0287650.s002.docx]

**S2 Table. List of raw analysis data**

Table. 1 List of data for different aerobic exercise. (C--- control)

| Aguilar-cordero 2019 | swimming | 6.41 | 3.68 | 70 |
| --- | --- | --- | --- | --- |
| Aguilar-cordero 2019 | C | 10.17 | 2.38 | 70 |
| Armstrong 2004 | cycling/walking/running | 6.33 | 3.67 | 9 |
| Armstrong 2004 | C | 13.33 | 7.66 | 10 |
| Buttner 2015 | swimming | 5.87 | 6.03 | 27 |
| Buttner 2015 | C | 8.52 | 5.43 | 29 |
| Coll 2019 | cycling/walking/running | 4.8 | 3.7 | 192 |
| Coll 2019 | C | 5.4 | 4.1 | 387 |
| Costa 2009 | yoga | 8.6 | 4.71 | 46 |
| Costa 2009 | C | 9 | 5.61 | 42 |
| Daley 2008 | dance | 13.1 | 5.2 | 16 |
| Daley 2008 | C | 14.3 | 5.4 | 15 |
| Daley 2015 | other sports | 12.51 | 5.46 | 47 |
| Daley 2015 | C | 14.67 | 4.86 | 47 |
| Forsyth 2017 | swimming | 11.8 | 6.1 | 11 |
| Forsyth 2017 | C | 12.7 | 4.2 | 11 |
| Haruna 2013 | swimming | 3.6 | 4.2 | 48 |
| Haruna 2013 | C | 4.1 | 3.4 | 47 |
| Heh 2018 | other sports | 10.2 | 3.6 | 63 |
| Heh 2018 | C | 12.7 | 3.9 | 63 |
| Keller 2014 | yoga | 7.05 | 5.36 | 39 |
| Keller 2014 | C | 7.8 | 5.05 | 54 |
| Lewis 2014 | other sports | 4.69 | 3.89 | 61 |
| Lewis 2014 | C | 7.02 | 4.64 | 63 |
| Mohammadi 2014 | other sports | 6.58 | 4.63 | 38 |
| Mohammadi 2014 | C | 6.5 | 5.12 | 36 |
| Norman 2010 | other sports | 5.47 | 5.11 | 62 |
| Norman 2010 | C | 6.75 | 5.11 | 73 |
| Özkan 2020 | dance | 7.29 | 1.67 | 40 |
| Özkan 2020 | C | 12.54 | 2.65 | 40 |
| Teychenne 2020 | other sports | 12.4 | 6.7 | 13 |
| Teychenne 2020 | C | 16.8 | 3.4 | 18 |
| Robichaud 2009 | yoga | 18.08 | 3.28 | 25 |
| Robichaud 2009 | C | 18.39 | 3.68 | 23 |
| Shelton 2015 | swimming | 3 | 1 | 3 |
| Shelton 2015 | C | 8 | 6 | 3 |
| Surkan 2012 | cycling/walking/running | 13.3 | 12.76 | 203 |
| Surkan 2012 | C | 15.3 | 12.76 | 200 |
| Thiruppathi 2014 | other sports | 4.95 | 0.68 | 20 |
| Thiruppathi 2014 | C | 7.52 | 0.51 | 21 |
| Yang&Chen 2017 | yoga | 7.6 | 4.71 | 60 |
| Yang&Chen 2017 | C | 7.18 | 4.54 | 62 |
| Ren Wei 2019 | cycling/walking/running | 9.94 | 2.32 | 19 |
| Ren Wei 2019 | C | 11.42 | 2.03 | 19 |
| LiLi 2019 | yoga | 9.68 | 2.14 | 50 |
| LiLi 2019 | C | 11.58 | 2.31 | 50 |
| Yan Feng 2019 | dance | 6.99 | 2.34 | 101 |
| Yan Feng 2019 | C | 8.21 | 3.32 | 111 |
| Huang Li 2003 | other sports | 7 | 4.6 | 39 |
| Huang Li 2003 | C | 5.97 | 5 | 31 |

Table. 2 List of data for different exercise intensity-duration. (C--- control)

| Aguilar-cordero 2019 | Moderate (35~45min) | 6.41 | 3.68 | 70 |
| --- | --- | --- | --- | --- |
| Aguilar-cordero 2019 | C | 10.17 | 2.38 | 70 |
| Armstrong 2004 | Moderate (35~45min) | 6.33 | 3.67 | 9 |
| Armstrong 2004 | C | 13.33 | 7.66 | 10 |
| Buttner 2015 | High (20~30min) | 5.87 | 6.03 | 27 |
| Buttner 2015 | C | 8.52 | 5.43 | 29 |
| Coll 2019 | High (20~30min) | 4.8 | 3.7 | 192 |
| Coll 2019 | C | 5.4 | 4.1 | 387 |
| Dacosta 2009 | High (20~30min) | 8.6 | 4.71 | 46 |
| Dacosta 2009 | C | 9 | 5.61 | 42 |
| Daley 2008 | Low(50~60min) | 13.1 | 5.2 | 16 |
| Daley 2008 | C | 14.3 | 5.4 | 15 |
| Daley 2015 | Low(50~60min) | 12.51 | 5.46 | 47 |
| Daley 2015 | C | 14.67 | 4.86 | 47 |
| Forsyth 2017 | High (20~30min) | 11.8 | 6.1 | 11 |
| Forsyth 2017 | C | 12.7 | 4.2 | 11 |
| Haruna 2013 | Low(50~60min) | 3.6 | 4.2 | 48 |
| Haruna 2013 | C | 4.1 | 3.4 | 47 |
| Heh 2018 | Moderate (35~45min) | 10.2 | 3.6 | 63 |
| Heh 2018 | C | 12.7 | 3.9 | 63 |
| Keller 2014 | Low(50~60min) | 7.05 | 5.36 | 39 |
| Keller 2014 | C | 7.8 | 5.05 | 54 |
| Mohammadi 2014 | Low(50~60min) | 6.58 | 4.63 | 38 |
| Mohammadi 2014 | C | 6.5 | 5.12 | 36 |
| Norman 2010 | Low(50~60min) | 5.47 | 5.11 | 62 |
| Norman 2010 | C | 6.75 | 5.11 | 73 |
| Özkan 2020 | Moderate (35~45min) | 7.29 | 1.67 | 40 |
| Özkan 2020 | C | 12.54 | 2.65 | 40 |
| Teychenne 2020 | Low(50~60min) | 12.4 | 6.7 | 13 |
| Teychenne 2020 | C | 16.8 | 3.4 | 18 |
| Saeedi 2010 | Moderate (35~45min) | 13.11 | 0.81 | 20 |
| Saeedi 2010 | C | 17.74 | 1.21 | 20 |
| Shelton 2015 | High (20~30min) | 3 | 1 | 3 |
| Shelton 2015 | C | 8 | 6 | 3 |
| Surkan 2012 | Low(50~60min) | 13.3 | 12.76 | 203 |
| Surkan 2012 | C | 15.3 | 12.76 | 200 |
| Thiruppathi 2014 | C | 7.52 | 0.51 | 21 |
| Yang&Chen 2017 | Low(50~60min) | 7.6 | 4.71 | 60 |
| Yang&Chen 2017 | 0 | 7.18 | 4.54 | 62 |
| Ren Wei 2019 | High (20~30min) | 9.94 | 2.32 | 19 |
| Ren Wei 2019 | C | 11.42 | 2.03 | 19 |
| Huang Li 2003 | Low(50~60min) | 7 | 4.6 | 39 |
| Huang Li 2003 | C | 5.97 | 5 | 31 |

Table.3 List of data for different exercise frequency. (C--- control)

| Aguilar-cordero 2019 | 3~4 times/week | 6.41 | 3.68 | 70 |
| --- | --- | --- | --- | --- |
| Aguilar-cordero 2019 | C | 10.17 | 2.38 | 70 |
| Armstrong 2004 | 3~4 times/week | 6.33 | 3.67 | 9 |
| Armstrong 2004 | C | 13.33 | 7.66 | 10 |
| Coll 2019 | 1~2 times/week | 4.8 | 3.7 | 192 |
| Coll 2019 | C | 5.4 | 4.1 | 387 |
| Costa 2009 | 1~2 times/week | 8.6 | 4.71 | 46 |
| Costa 2009 | C | 9 | 5.61 | 42 |
| Daley 2008 | 1~2 times/week | 13.1 | 5.2 | 16 |
| Daley 2008 | C | 14.3 | 5.4 | 15 |
| Daley 2015 | 1~2 times/week | 12.51 | 5.46 | 47 |
| Daley 2015 | C | 14.67 | 4.86 | 47 |
| Forsyth 2017 | 1~2 times/week | 11.8 | 6.1 | 11 |
| Forsyth 2017 | C | 12.7 | 4.2 | 11 |
| Haruna 2013 | 5~6 times/week | 3.6 | 4.2 | 48 |
| Haruna 2013 | C | 4.1 | 3.4 | 47 |
| Heh 2018 | 3~4 times/week | 10.2 | 3.6 | 63 |
| Heh 2018 | C | 12.7 | 3.9 | 63 |
| Keller 2014 | 5~6 times/week | 7.05 | 5.36 | 39 |
| Keller 2014 | C | 7.8 | 5.05 | 54 |
| Lewis 2014 | 5~6 times/week | 4.69 | 3.89 | 61 |
| Lewis 2014 | C | 7.02 | 4.64 | 63 |
| Mohammadi 2014 | 5~6 times/week | 6.58 | 4.63 | 38 |
| Mohammadi 2014 | C | 6.5 | 5.12 | 36 |
| Norman 2010 | 5~6 times/week | 5.47 | 5.11 | 62 |
| Norman 2010 | C | 6.75 | 5.11 | 73 |
| Özkan 2020 | 3~4 times/week | 7.29 | 1.67 | 40 |
| Özkan 2020 | C | 12.54 | 2.65 | 40 |
| Teychenne 2020 | 5~6 times/week | 12.4 | 6.7 | 13 |
| Teychenne 2020 | C | 16.8 | 3.4 | 18 |
| Robichaud 2009 | 5~6 times/week | 18.08 | 3.28 | 25 |
| Robichaud 2009 | C | 18.39 | 3.68 | 23 |
| Saeedi 2010 | 3~4 times/week | 13.11 | 0.81 | 20 |
| Saeedi 2010 | C | 17.74 | 1.21 | 20 |
| Shelton 2015 | 5~6 times/week | 3 | 1 | 3 |
| Shelton 2015 | C | 8 | 6 | 3 |
| Surkan 2012 | 5~6 times/week | 13.3 | 12.76 | 203 |
| Surkan 2012 | C | 15.3 | 12.76 | 200 |
| Thiruppathi 2014 | 5~6 times/week | 4.95 | 0.68 | 20 |
| Thiruppathi 2014 | C | 7.52 | 0.51 | 21 |
| Yang&Chen 2017 | 5~6 times/week | 7.6 | 4.71 | 60 |
| Yang&Chen 2017 | C | 7.18 | 4.54 | 62 |
| Ren Wei 2019 | 5~6 times/week | 9.94 | 2.32 | 19 |
| Ren Wei 2019 | C | 11.42 | 2.03 | 19 |
| LiLi 2019 | 5~6 times/week | 9.68 | 2.14 | 50 |
| LiLi 2019 | C | 11.58 | 2.31 | 50 |
| Yan Feng 2019 | 5~6 times/week | 6.99 | 2.34 | 101 |
| Yan Feng 2019 | C | 8.21 | 3.32 | 111 |
| Huang Li 2003 | 5~6 times/week | 7 | 4.6 | 39 |
| Huang Li 2003 | C | 5.97 | 5 | 31 |
