# Supplementary material for: Effectiveness of aerobic exercise in the prevention and treatment of postpartum depression: Meta-analysis and network meta-analysis
Source: PLoS One. 2023 Nov 29;18(11):e0287650. doi: 10.1371/journal.pone.0287650 (PMC10686497; doi:10.1371/journal.pone.0287650)
Supplement: S3 Table — (DOCX) [file pone.0287650.s003.docx]

**S3 Table.** **Complete league table**

Table. 1 The effect of different aerobic exercise programs on improving PPD symptom. (A = control, B = cycling/walking/running, C = dance, D = other sports, E = yoga, F = swimming)

| D | D | E | B | A | C | F |
| --- | --- | --- | --- | --- | --- | --- |
|  | D | 0.57 (-1.83,2.96) | 1.15 (-0.91,3.21) | 1.22 (-1.20,3.64) | 2.22 (0.00,4.44) | 2.89 (1.15,4.63) |
| E | -0.57 (-2.96,1.83) | E | 0.58 (-1.40,2.56) | 0.65 (-1.68,2.99) | 1.65 (-0.49,3.79) | 2.32 (0.68,3.96) |
| B | -1.15 (-3.21,0.91) | -0.58 (-2.56,1.40) | B | 0.07 (-1.93,2.07) | 1.07 (-0.70,2.84) | 1.74 (0.63,2.85) |
| A | -1.22 (-3.64,1.20) | -0.65 (-2.99,1.68) | -0.07 (-2.07,1.93) | A | 1.00 (-1.16,3.16) | 1.67 (0.01,3.33) |
| C | -2.22 (-4.44,-0.00) | -1.65 (-3.79,0.49) | -1.07 (-2.84,0.70) | -1.00 (-3.16,1.16) | C | 0.67 (-0.71,2.05) |
| F | -2.89 (-4.63,-1.15) | -2.32 (-3.96,-0.68) | -1.74 (-2.85,-0.63) | -1.67 (-3.33,-0.01) | -0.67 (-2.05,0.71) | F |

Table. 2 The effect of different exercise intensity-duration on improving PPD symptoms. (A =control, B =High (20~30min), C= Moderate (35~45min), D = Low (50~60min)

| C | C | D | B | A |
| --- | --- | --- | --- | --- |
|  | C | 2.63 (1.21,4.05) | 2.96 (1.41,4.51) | 3.92 (3.01,4.83) |
| D | -2.63 (-4.05,-1.21) | D | 0.33 (-1.32,1.98) | 1.29 (0.20,2.38) |
| B | -2.96 (-4.51,-1.41) | -0.33 (-1.98,1.32) | B | 0.96 (-0.29,2.20) |
| A | -3.92 (-4.83,-3.01) | -1.29 (-2.38,-0.20) | -0.96 (-2.20,0.29) | A |

Table. 3 The effect of different exercise frequency on improving PPD symptoms. (A =control, B =1~2 times/week, C =3~4 times/week, D = 5~6 times/week)

| C | C | B | D | A |
| --- | --- | --- | --- | --- |
|  | C | 2.91 (1.83,3.99) | 3.28 (1.81,4.75) | 4.22 (3.31,5.12) |
| B | -2.91 (-3.99,-1.83) | B | 0.37 (-0.95,1.68) | 1.31 (0.70,1.91) |
| D | -3.28 (-4.75,-1.81) | -0.37 (-1.68,0.95) | D | 0.94 (-0.22,2.10) |
| A | -4.22 (-5.12,-3.31) | -1.31 (-1.91,-0.70) | -0.94 (-2.10,0.22) | A |
