# Supplementary material for: Effectiveness of aerobic exercise in the prevention and treatment of postpartum depression: Meta-analysis and network meta-analysis
Source: PLoS One. 2023 Nov 29;18(11):e0287650. doi: 10.1371/journal.pone.0287650 (PMC10686497; doi:10.1371/journal.pone.0287650)
Supplement: S1 File — (PDF) [file pone.0287650.s004.pdf]

UNIVERSITY of York
Centre for Reviews and Dissemination

Systematic review

This record cannot be edited, the ID is CRD42023398221

1. * Review title.

Give the title of the review in English

Effectiveness of Aerobic Exercise in the Prevention and Treatment of Postpartum Depression: Meta-analysis and Network Meta-analysis

2. Original language title.

For reviews in languages other than English, give the title in the original language. This will be displayed with the English language title.

Effectiveness of Aerobic Exercise in the Prevention and Treatment of Postpartum Depression: Meta-analysis and Network Meta-analysis

3. * Anticipated or actual start date.

Give the date the systematic review started or is expected to start.

01/12/2022

4. * Anticipated completion date.

Give the date by which the review is expected to be completed.

20/03/2023

5. * Stage of review at time of this submission.

This field uses answers to initial screening questions. It cannot be edited until after registration.

Tick the boxes to show which review tasks have been started and which have been completed.

Update this field each time any amendments are made to a published record.

The review has not yet started: No

Review stage	Started	Completed
Preliminary searches	Yes	Yes
Piloting of the study selection process	Yes	Yes
Formal screening of search results against eligibility criteria	Yes	Yes
Data extraction	No	No
Risk of bias (quality) assessment	No	No
Data analysis	No	No

Provide any other relevant information about the stage of the review here.

6. * Named contact.

The named contact is the guarantor for the accuracy of the information in the register record. This may be any member of the review team.

Hao Xu

Email salutation (e.g. "Dr Smith" or "Joanne") for correspondence:
Ms Xu

7. * Named contact email.

Give the electronic email address of the named contact.

megan.xu@foxmail.com

8. Named contact address

PLEASE NOTE this information will be published in the PROSPERO record so please do not enter private information, i.e. personal home address

Give the full institutional/organisational postal address for the named contact.

School of Physical Education, China University of Geosciences, Wuhan, 430074, China

9. Named contact phone number.

Give the telephone number for the named contact, including international dialling code.

13409699375

10. * Organisational affiliation of the review.

Full title of the organisational affiliations for this review and website address if available. This field may be completed as 'None' if the review is not affiliated to any organisation.

China University of Geosciences

Organisation web address:

11. * Review team members and their organisational affiliations.

Give the personal details and the organisational affiliations of each member of the review team. Affiliation refers to groups or organisations to which review team members belong.
NOTE: email and country now MUST be entered for each person, unless you are amending a published record.

Ms Hao Xu. China University of Geosciences
Mr xiubing Wang. China University of Geosciences
Mr Jiahui Yang. China University of Geosciences

12. * Funding sources/sponsors.

Details of the individuals, organizations, groups, companies or other legal entities who have funded or sponsored the review.

This work was financially supported by the "Outstanding Talents Cultivation Fund" of the Central University Basic Scientific Research Fund

Grant number(s)
State the funder, grant or award number and the date of award
Grant no. CUG150607

13. * Conflicts of interest.

List actual or perceived conflicts of interest (financial or academic).

None

14. Collaborators.

Give the name and affiliation of any individuals or organisations who are working on the review but who are not listed as review team members. **NOTE: email and country must be completed for each person, unless you are amending a published record.**

15. * Review question.

State the review question(s) clearly and precisely. It may be appropriate to break very broad questions down into a series of related more specific questions. Questions may be framed or refined using PI(E)COS or similar where relevant.

Aerobic exercise is medicine for the prevention and treatment of postpartum depression, however, little is known about the most efficacious amount of exercise and the exercise protocol. This review aims to provide recommendations for the design and implementation of exercise prescription in this population.

16. * Searches.

State the sources that will be searched (e.g. Medline). Give the search dates, and any restrictions (e.g. language or publication date). Do NOT enter the full search strategy (it may be provided as a link or attachment below.)

Search strategy was conducted in these domestic and international databases: China National Knowledge Infrastructure (CNKI), Wanfang Database, MEDLINE, ScienceDirect, PubMed. We searched for all randomized controlled trial studies on exercise interventions for PPD, and identified keywords. The following complete search strategy was employed: ((postpartum depression [Title/Abstract] OR postnatal depression [Title/Abstract]) AND (Maternal depression [Title/Abstract] OR Maternal depressive symptoms [Title/Abstract]). The interventions include exercise OR train OR physical activity OR aerobic exercise were selected. The last search was performed on January 2023. See Annex Box 1 for specific search strategies.

17. URL to search strategy.

Upload a file with your search strategy, or an example of a search strategy for a specific database, (including the keywords) in pdf or word format. In doing so you are consenting to the file being made publicly accessible.

Or provide a URL or link to the strategy. Do NOT provide links to your search **results**.

https://www.crd.york.ac.uk/PROSPEROFILES/398221_STRATEGY_20230209.pdf

Do not make this file publicly available until the review is complete

18. * Condition or domain being studied.

Give a short description of the disease, condition or healthcare domain being studied in your systematic review.

As a common complication of childbirth, postpartum depression (PPD) is defined as an obvious depressive symptom or a typical depressive episode within 1 to 12 months after delivery (Pritchett et al., 2020). PPD represents a significant public health problem that threatens the physical and mental health of mothers and babies (Haviland et al., 2022). According to 2021 estimates, approximately 13 million women around the world are diagnosed with PPD each year with prevalence is between 0.5% and 63.3% (Shi et al., 2021; Abenova et al., 2022). PPD has high incidence rate and low treatment rate (90% of the patients are untreated) which brings a heavy burden to families and society (Albright et al., 2014). Traditional treatment for PPD mainly consists of psychological and medication interventions. The economic cost of psychotherapy and the side effects of antidepressant medications have led to poor adherence and poor treatment outcomes.

19. * Participants/population.

Specify the participants or populations being studied in the review. The preferred format includes details of both inclusion and exclusion criteria.

Participants: patients /potential patients with postpartum depression
Inclusion criteria: the subjects included women with diagnosed PPD or at risk for PPD during the perinatal period
Exclusion criteria: the population with depression except for pregnant women

20. * Intervention(s), exposure(s).

Give full and clear descriptions or definitions of the interventions or the exposures to be reviewed. The preferred format includes details of both inclusion and exclusion criteria.

Exercise interventions：As a new “prescription tool”, exercise interventions are not only an important non-pharmacological method in treating postpartum depression, but also effective in preventing this disorder. Aerobic exercise as a common type of exercise for postpartum depression management. Current evidence supports that PPD can be effectively prevented and treated through exercise due to the postpartum-specific health outcomes including less urinary stress incontinence, less lactation-induced bone loss, reducing postpartum weight retention, and less anxiety and depression (Garnaes et al., 2019). It is widely recognized for the advantages of high practical operability and safety.
Inclusion criteria: the exercise intervention type in the experimental group was aerobic exercise
Exclusion criteria: Interventions were only psychotherapy and medication

21. * Comparator(s)/control.

Where relevant, give details of the alternatives against which the intervention/exposure will be compared (e.g. another intervention or a non-exposed control group). The preferred format includes details of both inclusion and exclusion criteria.

All control groups were usual care

22. * Types of study to be included.

Give details of the study designs (e.g. RCT) that are eligible for inclusion in the review. The preferred format includes both inclusion and exclusion criteria. If there are no restrictions on the types of study, this should be stated.

A randomized controlled trial (RCT) types of study to be included.
Inclusion criteria: RCTs
Exclusion criteria: Non-randomized controlled experimental study

23. Context.

Give summary details of the setting or other relevant characteristics, which help define the inclusion or exclusion criteria.

Inclusion criteria: (1) the analysis type in the literature is a randomized controlled trial (RCT); (2) the subjects included women with diagnosed PPD or at risk for PPD during the perinatal period; (3) the exercise intervention type in the experimental group was aerobic exercise; (4) the Edinburgh Postpartum Depression Symptom Scale (EPDS) was used for the diagnosis of PPD in the included studies.
Exclusion criteria: (1) review articles; (2) the population with depression except for pregnant women; (3) the non-exercise interventions. (4) Screening for postpartum depressive symptoms using other scales: The World Health Organization 5 Physical and Mental Health Indicators (WHO-5), Pittsburgh Sleep Quality Index (PSQI), Beck Depression Inventory (BDI), Hamilton Anxiety Inventory (HAMA); (5) inadequate trial data; (6) animal testing.

24. * Main outcome(s).

Give the pre-specified main (most important) outcomes of the review, including details of how the outcome is defined and measured and when these measurement are made, if these are part of the review inclusion criteria.

We performed meta-analysis of the mean EPDS scores of the experimental and control groups before and after the aerobic exercise intervention

Measures of effect
We performed meta-analysis of the mean EPDS scores of the experimental and control groups before and after the aerobic exercise intervention using RevMan 5.3 software. Effect values were counted using the mean difference (MD) and 95% confidence interval (CI) because the outcome indicators of all included studies were EPDS scale scores, which are continuous variables of the same measure. When the 95% CL included 0, it indicated no statistical significance. If $I^2 \le 50\%$ or $p > 0.1$ indicates less heterogeneity, a fixed-effects model was chosen. On the contrary $I^2 > 50\%$ or $p \le 0.1$, it means that the heterogeneity is large, and a random-effects model should be used after analyzing the source of heterogeneity (e.g., sensitivity analysis or subgroup analysis).

25. * Additional outcome(s).

List the pre-specified additional outcomes of the review, with a similar level of detail to that required for main outcomes. Where there are no additional outcomes please state ‘None’ or ‘Not applicable’ as appropriate to the review

Measures of effect

26. * Data extraction (selection and coding).

Describe how studies will be selected for inclusion. State what data will be extracted or obtained. State how this will be done and recorded.

When heterogeneity is high, we need to go through a subgroup analysis with the aim of exploring the sources of heterogeneity and deriving a better protocol for the exercise intervention. After comparing the exercise guidelines of the studies, striking differences were found in factors such as timing of intervention, supervision or not, and form of exercise (individual or team), with significantly less heterogeneity between studies when grouped by these factors. However, subgroup heterogeneity remained high for timing of exercise intervention. Effect values were counted using the mean difference (MD) and 95% confidence interval (CI) because the outcome indicators of all included studies were EPDS scale scores, which are continuous variables of the same measure. When the 95% CL included 0, it indicated no statistical significance. If $I^2 \leq 50\%$ or $p > 0.1$ indicates less heterogeneity, a fixed-effects model was chosen. On the contrary $I^2 > 50\%$ or $p \leq 0.1$, it means that the heterogeneity is large, and a random-effects model should be used after analyzing the source of heterogeneity (e.g., sensitivity analysis or subgroup analysis).When heterogeneity is high, we need to go through a subgroup analysis with the aim of exploring the sources of heterogeneity and deriving a better protocol for the exercise intervention. After comparing the exercise guidelines of the 26 studies, striking differences were found in factors such as timing of intervention, supervision or not, and form of exercise (individual or team), with significantly less heterogeneity between studies when grouped by these factors. However, subgroup heterogeneity remained high for timing of exercise intervention.

27. * Risk of bias (quality) assessment.

State which characteristics of the studies will be assessed and/or any formal risk of bias/quality assessment tools that will be used.

According to the preliminary risk assessment for publication bias as recommended by the Cochrane Collaboration, the following parameters were included: adequate random sequence generation, allocation concealment, blinding of participants and personnel, blinding of outcome assessment, incomplete outcome data, selective reporting and other biases.

28. * Strategy for data synthesis.

Describe the methods you plan to use to synthesise data. This **must not be generic text** but should be **specific to your review** and describe how the proposed approach will be applied to your data.
If meta-analysis is planned, describe the models to be used, methods to explore statistical heterogeneity, and software package to be used.

We performed meta-analysis of the mean EPDS scores of the experimental and control groups before and after the aerobic exercise intervention using RevMan 5.3 software. Effect values were counted using the mean difference (MD) and 95% confidence interval (CI) because the outcome indicators of all included studies were EPDS scale scores, which are continuous variables of the same measure. When the 95% CL included 0, it indicated no statistical significance. If $I^2 \leq 50\%$ or $p > 0.1$ indicates less heterogeneity, a fixed-effects model was chosen. On the contrary $I^2 > 50\%$ or $p \leq 0.1$, it means that the heterogeneity is large, and a random-effects model should be used after analyzing the source of heterogeneity (e.g., sensitivity analysis or subgroup analysis).

29. * Analysis of subgroups or subsets.

State any planned investigation of 'subgroups'. Be clear and specific about which type of study or participant will be included in each group or covariate investigated. State the planned analytic approach.

when heterogeneity is high, we need to go through a subgroup analysis with the aim of exploring the sources of heterogeneity and deriving a better protocol for the exercise intervention. After comparing the exercise guidelines of the 26 studies, striking differences were found in factors such as timing of intervention, supervision or not, and form of exercise (individual or team), with significantly less heterogeneity between studies when grouped by these factors.

However, subgroup heterogeneity remained high for timing of exercise intervention。
Further observation of the intervention elements in this subgroup revealed that all studies in this subgroup also had differences in factors such as aerobic exercise program and amount of exercise (intensity, duration, frequency, and total duration of intervention), due to the large number of influencing factors, the heterogeneity was high, Stata 16.0 software was used to perform a network meta-analysis and draw an evidence network diagram. As there was no closed loop in the evidence network diagram of this study, no inconsistency test was required and comparisons were made directly,

and drew the league table (the data in the table represents the MD values and 95% CI values for direct two-by-two comparisons of different interventions. When MD<0, the "column" treatment measures were superior to the "row", and vice versa. When 95% CI don't included 0 , it indicated no statistical significance (p<0.05), and vice versa. Then, the Surface Under The Cumulative Ranking (SUCRA) of each intervention was calculated, and the higher the probability, the better the intervention effect.

30. * Type and method of review.

Select the type of review, review method and health area from the lists below.

Type of review

Cost effectiveness	No
Diagnostic	No
Epidemiologic	No
Individual patient data (IPD) meta-analysis	No
Intervention	No
Living systematic review	No
Meta-analysis	Yes
Methodology	No
Narrative synthesis	No
Network meta-analysis	Yes
Pre-clinical	No
Prevention	No
Prognostic	No
Prospective meta-analysis (PMA)	No
Review of reviews	No
Service delivery	No
Synthesis of qualitative studies	No
Systematic review	Yes
Other	No

Health area of the review

Alcohol/substance misuse/abuse	No
Blood and immune system	No
Cancer	No
Cardiovascular	No
Care of the elderly	No

Child health	No
Complementary therapies	No
COVID-19	No
Crime and justice	No
Dental	No
Digestive system	No
Ear, nose and throat	No
Education	No
Endocrine and metabolic disorders	No
Eye disorders	No
General interest	No
Genetics	No
Health inequalities/health equity	No
Infections and infestations	No
International development	No
Mental health and behavioural conditions	Yes
Musculoskeletal	No
Neurological	No
Nursing	No
Obstetrics and gynaecology	Yes
Oral health	No
Palliative care	No
Perioperative care	No
Physiotherapy	Yes
Pregnancy and childbirth	No
Public health (including social determinants of health)	Yes
Rehabilitation	No
Respiratory disorders	No
Service delivery	No
Skin disorders	No

Social care	Yes
Surgery	No
Tropical Medicine	No
Urological	No
Wounds, injuries and accidents	No
Violence and abuse	No

31. Language.

Select each language individually to add it to the list below, use the bin icon to remove any added in error.

English

There is not an English language summary

32. * Country.

Select the country in which the review is being carried out. For multi-national collaborations select all the countries involved.

China

33. Other registration details.

Name any other organisation where the systematic review title or protocol is registered (e.g. Campbell, or The Joanna Briggs Institute) together with any unique identification number assigned by them.
If extracted data will be stored and made available through a repository such as the Systematic Review Data Repository (SRDR), details and a link should be included here. If none, leave blank.

34. Reference and/or URL for published protocol.

If the protocol for this review is published provide details (authors, title and journal details, preferably in Vancouver format)

No I do not make this file publicly available until the review is complete

35. Dissemination plans.

Do you intend to publish the review on completion?

No

36. Keywords.

Give words or phrases that best describe the review. Separate keywords with a semicolon or new line. Keywords help PROSPERO users find your review (keywords do not appear in the public record but are included in searches). Be as specific and precise as possible. Avoid acronyms and abbreviations unless these are in wide use.

aerobic exercise; postpartum depression; exercise guidelines; amount of exercise; meta-analysis

37. Details of any existing review of the same topic by the same authors.

If you are registering an update of an existing review give details of the earlier versions and include a full bibliographic reference, if available.

38. * Current review status.

Update review status when the review is completed and when it is published.
New registrations must be ongoing so this field is not editable for initial submission.

Review_Ongoing

39. Any additional information.

Provide any other information relevant to the registration of this review.

40. Details of final report/publication(s) or preprints if available.

Leave empty until publication details are available OR you have a link to a preprint (NOTE: this field is not editable for initial submission).
List authors, title and journal details preferably in Vancouver format.
