# Supplementary material for: Effectiveness of aerobic exercise in the prevention and treatment of postpartum depression: Meta-analysis and network meta-analysis
Source: PLoS One. 2023 Nov 29;18(11):e0287650. doi: 10.1371/journal.pone.0287650 (PMC10686497; doi:10.1371/journal.pone.0287650)
Supplement: S2 File — (DOCX) [file pone.0287650.s005.docx]

**S2 File. Search strategy**

PubMed search strategy as an example.

#1 postpartum depression [MeSH ] OR postnatal depression [Title/Abstract] OR

depression, postnatal [Title/Abstract] OR post-partum depression [Title/Abstract] OR depression, post-partum [Title/Abstract] OR postpartum depression [Title/Abstract] OR post-natal depression [Title/Abstract] OR Depression, post-Natal [Title/Abstract] OR post natal depression[Title/Abstract]

#2 exercises [MeSH] OR physical activity [Title/Abstract] OR activities, Physical [Title/Abstract] OR activity, physical [Title/Abstract] OR physical activities [Title/Abstract] OR exercise, physical [Title/Abstract] OR exercises, physical [Title/Abstract] OR physical exercise [Title/Abstract] OR Physical Exercises [Title/Abstract] OR acute exercise [Title/Abstract] OR acute exercises [Title/Abstract])) OR exercise, acute [Title/Abstract] OR exercises,acute [Title/Abstract] OR exercises, isometric [Title/Abstract] OR isometric exercises [Title/Abstract] OR exercise, aerobic [Title/Abstract] OR aerobic exercise [Title/Abstract] OR aerobic exercises [Title/Abstract] OR exercise training [Title/Abstract] OR exercise training [Title/Abstract] OR training, exercise [Title/Abstract] OR training, exercise [Title/Abstract])

#3 #1 AND #2

#4 randomized controlled trial [pt] OR allocation, random[Title/Abstract] OR randomization [pt] OR clinical trials, randomized [tiab] OR trials, randomized clinical [tiab] OR controlled clinical trials, randomized [tiab] NOT animanls [mh]

#5 #3 AND #4
