# Supplementary material for: Effectiveness of aerobic exercise in the prevention and treatment of postpartum depression: Meta-analysis and network meta-analysis
Source: PLoS One. 2023 Nov 29;18(11):e0287650. doi: 10.1371/journal.pone.0287650 (PMC10686497; doi:10.1371/journal.pone.0287650)
Supplement: S3 File — (DOCX) [file pone.0287650.s006.docx]

**S3 File. Data analysis and coding process**

Table.5 Network plots and surface under cumulative ranking curves (SUCRA**)**

|  |
| --- |
| /__ / ____/ / ____/ |
| ___/ / /___/ / /___/ 16.0 Copyright 1985-2019 StataCorp LLC |
| Statistics/Data Analysis StataCorp |
| 4905 Lakeway Drive |
| Special Edition College Station, Texas 77845 USA |
| 800-STATA-PC http://www.stata.com |
| 979-696-4600 stata@stata.com |
| 979-696-4601 (fax) |
| Single-user Stata perpetual license: |
| Serial number: 12345678910 |
| Licensed to: 123 |
| 123 |
| Notes: |
| 1. Unicode is supported; see help unicode_advice. |
| 2. Maximum number of variables is set to 5000; see help set_maxvar. |
| running c:\ado\plus\profile.do ... |
| . *(4 variables, 50 observations pasted into data editor) |
| . clear |
| . *(5 variables, 50 observations pasted into data editor) |
| . network setup var3 var4 var5, study(var1) trt(var2) format(augment) md |
| Treatments used |
| A (reference): control |
| B: cycling/walking/running |
| C: dance |
| D: other sports |
| E: yoga |
| F: swimming |
| Measure mean difference |
| Standard deviation pooling: on |
| Studies |
| ID variable: var1 |
| Number used: 25 |
| Network information |
| Components: 1 (connected) |
| D.f. for inconsistency: 0 |
| D.f. for heterogeneity: 20 |
| Current data |
| Data format: augmented |
| Design variable: _design |
| Estimate variables: _y* |
| Variance variables: _S* |
| Command to list the data: list var1 _y* _S*, noo sepby(_design) |
| . network map |
| Graph command stored in F9 |
| . network meta c |
| Command is: mvmeta _y _S , bscovariance(exch 0.5) longparm suppress(uv mm) vars(_y_B _y_C _y_D _y_E _y_ |
| > F) |
| Note: using method reml |
| Note: using variables _y_B _y_C _y_D _y_E _y_F |
| Note: 25 observations on 5 variables |
| Note: variance-covariance matrix is proportional to .5*I(5)+.5*J(5,5,1) |
| initial: log likelihood = -30.848024 |
| rescale: log likelihood = -30.848024 |
| rescale eq: log likelihood = -30.848024 |
| Iteration 0: log likelihood = -30.848024 |
| Iteration 1: log likelihood = -30.050701 |
| Iteration 2: log likelihood = -30.034176 |
| Iteration 3: log likelihood = -30.034107 |
| Iteration 4: log likelihood = -30.034107 |
| Multivariate meta-analysis |
| Variance-covariance matrix = proportional .5*I(5)+.5*J(5,5,1) |
| Method = reml Number of dimensions = 5 |
| Restricted log likelihood = -30.034107 Number of observations = 25 |
| Coef. Std. Err. z P>z [95% Conf. Interval] |
| _y_B |
| _cons -.4625774 .4053334 -1.14 0.254 -1.257016 .3318615 |
| _y_C |
| _cons -.9905894 .4653524 -2.13 0.033 -1.902663 -.0785155 |
| _y_D |
| _cons -.6958888 .2888656 -2.41 0.016 -1.262055 -.1297226 |
| _y_E |
| _cons -.213448 .3532371 -0.60 0.546 -.9057801 .478884 |
| _y_F |
| _cons -.5514346 .3825878 -1.44 0.149 -1.301293 .1984237 |
|  |
| Estimated between-studies SDs and correlation matrix: |
| SD _y_B _y_C _y_D _y_E _y_F |
| _y_B .75786233 1 . . . . |
| _y_C .75786233 .5 1 . . . |
| _y_D .75786233 .5 .5 1 . . |
| _y_E .75786233 .5 .5 .5 1 . |
| _y_F .75786233 .5 .5 .5 .5 1 |
| mvmeta command stored as F9 |
| . network rank min, all zero reps(5000) gen(prob) |
| Command is: mvmeta, noest pbest(min in 1, zero id(var1) all reps(5000) gen(prob) stripprefix(_y_) zerona |
| > me(A) rename(A = control, B = cycling/walking/running, C = dance, D = other sports, E = yoga, F = swimming)) |
| Estimated probabilities (%) of each treatment being the best (and other ranks) |
| - assuming the minimum parameter is the best |
| - using 5000 draws |
| - allowing for parameter uncertainty |
| Treatment |
| var1 and Rank control、cycling/walking/running、 dance、 other sports、 yoga 、swimming |
| Aguilar-cordero 2019 |
| Best 0.0 10.3 56.3 18.4 2.2 12.8 |
| 2nd 0.0 17.2 20.6 33.6 6.8 21.8 |
| 3rd 0.3 21.4 11.7 27.8 13.3 25.5 |
| 4th 7.1 25.0 7.5 15.2 23.4 21.8 |
| 5th 36.0 15.3 2.7 4.4 29.4 12.2 |
| Worst 56.6 10.8 1.3 0.5 25.0 5.9 |
| mvmeta command is stored in F9 |
| . sucra prob*, lab(A B C D E F) |
| Treatment Relative Ranking of Model 1 |
|  |
| Treatm~t SUCRA PrBest MeanRank |
| - |
| A 4.2 0.0 5.5 |
| B 54.4 10.3 3.5 |
| C 86.9 56.3 1.8 |
| D 56.6 18.4 2.6 |
| E 24.3 2.2 4.5 |
| F 73.3 12.8 3.2 |
|  |
| . netleague,lab(A B C D E F)sort(C D F B E A) |
| Warning: The existing dataset is stored as a temporary file |
| Warning: To save any changes applied at this temporary file in a specific directory you need to use the |
| > 'Save as' menu |
| The league table has been stored at the end of the dataset |
| . clear |
| . *(5 variables, 48 observations pasted into data editor) |
| . network setup var3 var4 var5, study(var1) trt(var2) format(augment) md |
| Treatments used |
| A (reference): control |
| B: 1~2 times/week |
| C: 3~4 times/week |
| D: 5~6 times/week |
| Measure mean difference |
| Standard deviation pooling: on |
| Studies |
| ID variable: var1 |
| Number used: 24 |
| Network information |
| Components: 1 (connected) |
| D.f. for inconsistency: 0 |
| D.f. for heterogeneity: 22 |
| Current data |
| Data format: augmented |
| Design variable: _design |
| Estimate variables: _y* |
| Variance variables: _S* |
| Command to list the data: list var1 _y* _S*, noo sepby(_design) |
| . network meta c |
| Command is: mvmeta _y _S , bscovariance(exch 0.5) longparm suppress(uv mm) vars(_y_B _y_C _y_D) |
| Note: using method reml |
| Note: using variables _y_B _y_C _y_D |
| Note: 25 observations on 3 variables |
| Note: variance-covariance matrix is proportional to .5*I(3)+.5*J(3,3,1) |
| initial: log likelihood = -34.156079 |
| rescale: log likelihood = -34.156079 |
| rescale eq: log likelihood = -34.156079 |
| Iteration 0: log likelihood = -34.156079 |
| Iteration 1: log likelihood = -33.807369 |
| Iteration 2: log likelihood = -33.696949 |
| Iteration 3: log likelihood = -33.696876 |
| Iteration 4: log likelihood = -33.696876 |
| Multivariate meta-analysis |
| Variance-covariance matrix = proportional .5*I(3)+.5*J(3,3,1) |
| Method = reml Number of dimensions = 3 |
| Restricted log likelihood = -33.696876 Number of observations = 25 |
| Coef. Std. Err. z P>z [95% Conf. Interval] |
| _y_B |
| _cons -.2061387 .3848012 -0.54 0.592 -.9603352 .5480579 |
| _y_C |
| _cons -1.800315 .4020501 -4.48 0.000 -2.588318 -1.012311 |
| _y_D |
| _cons -.5032788 .2262542 -2.22 0.026 -.9467289 -.0598287 |
|  |
| Estimated between-studies SDs and correlation matrix: |
| SD _y_B _y_C _y_D |
| _y_B .81475018 1 . . |
| _y_C .81475018 .5 1 . |
| _y_D .81475018 .5 .5 1 |
| mvmeta command stored as F9 |
| . network rank min, all zero reps(5000) gen(prob |
| ) required |
| r(100); |
| . network rank min, all zero reps(5000) gen(prob) |
| Command is: mvmeta, noest pbest(min in 1, zero id(var1) all reps(5000) gen(prob) stripprefix(_y_) zerona |
| > me(A) rename(A = control, B = 1~2 times/week, C = 3~4 times/week, D = 5~6 times/week)) |
| Estimated probabilities (%) of each treatment being the best (and other ranks) |
| - assuming the minimum parameter is the best |
| - using 5000 draws |
| - allowing for parameter uncertainty |
| Treatment |
| var1 and Rank 0 6 61 62 |
| Aguilar-cordero 2019 |
| Best 0.0 0.3 99.5 0.2 |
| 2nd 0.3 25.0 0.5 74.3 |
| 3rd 29.5 46.1 0.0 24.5 |
| Worst 70.3 28.7 0.0 1.1 |
| mvmeta command is stored in F9 |
| . sucra prob*, lab(control ,1~2 times/week, 3~4 times/week,5~6 times/week) |
| Treatment Relative Ranking of Model 1 |
|  |
| Treatm~t SUCRA PrBest MeanRank |
| - |
| A (reference): control |
| B: 1~2 times/week |
| C: 3~4 times/week |
| D: 5~6 times/week |
| . sucra prob*, lab(A B C D) |
| Treatment Relative Ranking of Model 1 |
| Treatm~t SUCRA PrBest MeanRank |
| - |
| A 1.8 0.0 3.7 |
| B 41.3 0.3 3.0 |
| C 100 99.5 1.0 |
| D 56.8 0.2 2.3 |
| . netleague,lab(A B C D)sort(C D B A) |
| Warning: The existing dataset is stored as a temporary file |
| Warning: To save any changes applied at this temporary file in a specific directory you need to use the |
| > 'Save as' menu |
| The league table has been stored at the end of the dataset |
| . clear |
| . *(5 variables, 32 observations pasted into data editor) |
| . network setup var3 var4 var5, study(var1) trt(var2) format(augment) md |
| Treatments used |
| A (reference): control |
| B: 65%~74%HRR-15~30min |
| C: 50%~60%HRR-35~45min |
| D: 40%HRR-50~60min |
| Measure Standardised mean difference |
| Standard deviation pooling: on |
| Studies |
| ID variable: var1 |
| Number used: 16 |
| Network information |
| Components: 1 (connected) |
| D.f. for inconsistency: 0 |
| D.f. for heterogeneity: 14 |
| Current data |
| Data format: augmented |
| Design variable: _design |
| Estimate variables: _y* |
| Variance variables: _S* |
| Command to list the data: list var1 _y* _S*, noo sepby(_design) |
| . network meta c |
| Command is: mvmeta _y _S , bscovariance(exch 0.5) longparm suppress(uv mm) vars(_y_B _y_C _y_D) |
| Note: using method reml |
| Note: using variables _y_B _y_C _y_D |
| Note: 17 observations on 3 variables |
| Note: variance-covariance matrix is proportional to .5*I(3)+.5*J(3,3,1) |
| initial: log likelihood = -21.085442 |
| rescale: log likelihood = -21.085442 |
| rescale eq: log likelihood = -21.085442 |
| Iteration 0: log likelihood = -21.085442 |
| Iteration 1: log likelihood = -20.234199 |
| Iteration 2: log likelihood = -20.228073 |
| Iteration 3: log likelihood = -20.22806 |
| Iteration 4: log likelihood = -20.22806 |
| Multivariate meta-analysis |
| Variance-covariance matrix = proportional .5*I(3)+.5*J(3,3,1) |
| Method = reml Number of dimensions = 3 |
| Restricted log likelihood = -20.22806 Number of observations = 17 |
| Coef. Std. Err. z P>z [95% Conf. Interval] |
| _y_B |
| _cons -.4945461 .325435 -1.52 0.129 -1.132387 .1432948 |
| _y_C |
| _cons -1.532427 .3235393 -4.74 0.000 -2.166552 -.8983015 |
| _y_D |
| _cons -.2062195 .3370551 -0.61 0.541 -.8668353 .4543964 |
|  |
| Estimated between-studies SDs and correlation matrix: |
| SD _y_B _y_C _y_D |
| _y_B .702152 1 . . |
| _y_C .702152 .5 1 . |
| _y_D .702152 .5 .5 1 |
| mvmeta command stored as F9 |
| . network rank min, all zero reps(5000) gen(prob) |
| Command is: mvmeta, noest pbest(min in 1, zero id(var1) all reps(5000) gen(prob) stripprefix(_y_) zerona |
| > me(A) rename(A = control, B = 65%~74%HRR-15~30min, C = 50%~60%HRR-35~45min, D = 40%HRR-50~60min)) |
| Estimated probabilities (%) of each treatment being the best (and other ranks) |
| - assuming the minimum parameter is the best |
| - using 5000 draws |
| - allowing for parameter uncertainty |
| Treatment |
| var1 and Rank control、 65%~74%HRR-15~30min 、50%~60%HRR-35~45min 、40%HRR-50~60min |
| Aguilar-cordero 2019 |
| Best 0.0 1.6 98.3 0.1 |
| 2nd 1.6 69.5 1.7 27.2 |
| 3rd 29.0 24.0 0.0 47.0 |
| Worst 69.4 4.9 0.0 25.7 |
| mvmeta command is stored in F9 |
| . sucra prob*, lab(A B C D) |
| Treatment Relative Ranking of Model 1 |
|  |
| Treatm~t SUCRA PrBest MeanRank |
| - |
| A 2.5 0.0 3.7 |
| B 55 1.6 2.3 |
| C 100 98.3 1.0 |
| D 42.5 0.1 3.0 |
|  |
| . netleague,lab(A B C D)sort(C B D A) |
| Warning: The existing dataset is stored as a temporary file |
| Warning: To save any changes applied at this temporary file in a specific directory you need to use the |
| > 'Save as' menu |
| The league table has been stored at the end of the dataset |
| . CLEAR |
| command CLEAR not defined by CLEAR.ado |
| r(199); |
| . clear |
| . *(5 variables, 32 observations pasted into data editor) |
| . network setup var3 var4 var5, study(var1) trt(var2) format(augment) md |
| Treatments used |
| A (reference): 12-16week |
| B: 18-24week |
| C: 4-8week |
| D: control |
| Measure mean difference |
| Standard deviation pooling: on |
| Studies |
| ID variable: var1 |
| Number used: 16 |
| IDs with augmented reference arm: `"Buttner 2015"' `"Daley 2015"' `"Haruna 2013"' `"Heh 2018"' `"Kel |
| > ler 2014"' `"Lewis 2014"' `"Mohammadi 2014"' `"Norman 2010"' `"Teychenne 2020"' `"Surkan 2012"' |
| observations added: 0.001 |
| mean in augmented observations: study-specific mean |
| SD in augmented observations: study-specific within-arms SD |
| Network information |
| Components: 1 (connected) |
| D.f. for inconsistency: 0 |
| D.f. for heterogeneity: 13 |
| Current data |
| Data format: augmented |
| Design variable: _design |
| Estimate variables: _y* |
| Variance variables: _S* |
| Command to list the data: list var1 _y* _S*, noo sepby(_design) |
| . network meta c |
| Command is: mvmeta _y _S , bscovariance(exch 0.5) longparm suppress(uv mm) vars(_y_B _y_C _y_D) |
| Note: using method reml |
| Note: using variables _y_B _y_C _y_D |
| Note: 16 observations on 3 variables |
| Note: variance-covariance matrix is proportional to .5*I(3)+.5*J(3,3,1) |
| initial: log likelihood = -61.724609 |
| rescale: log likelihood = -61.724609 |
| rescale eq: log likelihood = -61.148299 |
| Iteration 0: log likelihood = -61.148299 |
| Iteration 1: log likelihood = -60.306408 |
| Iteration 2: log likelihood = -60.295349 |
| Iteration 3: log likelihood = -60.295316 |
| Iteration 4: log likelihood = -60.295316 |
| Multivariate meta-analysis |
| Variance-covariance matrix = proportional .5*I(3)+.5*J(3,3,1) |
| Method = reml Number of dimensions = 3 |
| Restricted log likelihood = -60.295316 Number of observations = 16 |
| Coef. Std. Err. z P>z [95% Conf. Interval] |
| _y_B |
| _cons -.0864903 .4362457 -0.20 0.843 -.9415162 .7685356 |
| _y_C |
| _cons -.0863588 .4365837 -0.20 0.843 -.9420472 .7693296 |
| _y_D |
| _cons .3154854 .3041175 1.04 0.300 -.2805739 .9115447 |
|  |
| Estimated between-studies SDs and correlation matrix: |
| SD _y_B _y_C _y_D |
| _y_B .66227975 1 . . |
| _y_C .66227975 .5 1 . |
| _y_D .66227975 .5 .5 1 |
| mvmeta command stored as F9 |
| . network rank min, all zero reps(5000) gen(prob) |
| Command is: mvmeta, noest pbest(min in 1, zero id(var1) all reps(5000) gen(prob) stripprefix(_y_) zerona |
| > me(A) rename(A = 12-16week, B = 18-24week, C = 4-8week, D = control)) |
| Estimated probabilities (%) of each treatment being the best (and other ranks) |
| - assuming the minimum parameter is the best |
| - using 5000 draws |
| - allowing for parameter uncertainty |
| Treatment |
| var1 and Rank 12-16 week 18-24 week 4-8 week control |
| Armstrong 2004 |
| Best 25.7 37.6 36.6 0.1 |
| 2nd 31.5 32.1 32.6 3.8 |
| 3rd 29.0 21.2 22.0 27.8 |
| Worst 13.9 9.0 8.7 68.4 |
| mvmeta command is stored in F9 |
| . sucra prob*, lab(A B C D) |
| Treatment Relative Ranking of Model 1 |
|  |
| Treatm~t SUCRA PrBest MeanRank |
| - |
| A 53.3 25.7 2.3 |
| B 72.2 37.6 2.0 |
| C 63.8 36.6 2.0 |
| D 10.1 0.1 3.6 |
|  |
| . netleague,lab(A B C D)sort(B C A D) |
| Warning: The existing dataset is stored as a temporary file |
| Warning: To save any changes applied at this temporary file in a specific directory you need to use the |
| > 'Save as' menu |
| The league table has been stored at the end of the dataset |
| . netleague,lab(A B C D)sort(B C A D) |
| Warning: The existing dataset is stored as a temporary file |
| Warning: To save any changes applied at this temporary file in a specific directory you need to use the |
| > 'Save as' menu |
| The league table has been stored at the end of the dataset |
| . clear |
| . |
