# Supplementary figures and images for: Effectiveness of aerobic exercise in the prevention and treatment of postpartum depression: Meta-analysis and network meta-analysis
Source: PLoS One. 2023 Nov 29;18(11):e0287650. doi: 10.1371/journal.pone.0287650 (PMC10686497; doi:10.1371/journal.pone.0287650)

**S4 File. List of sensitivity analysis data**

Table.1 List of sensitivity analysis data.

**
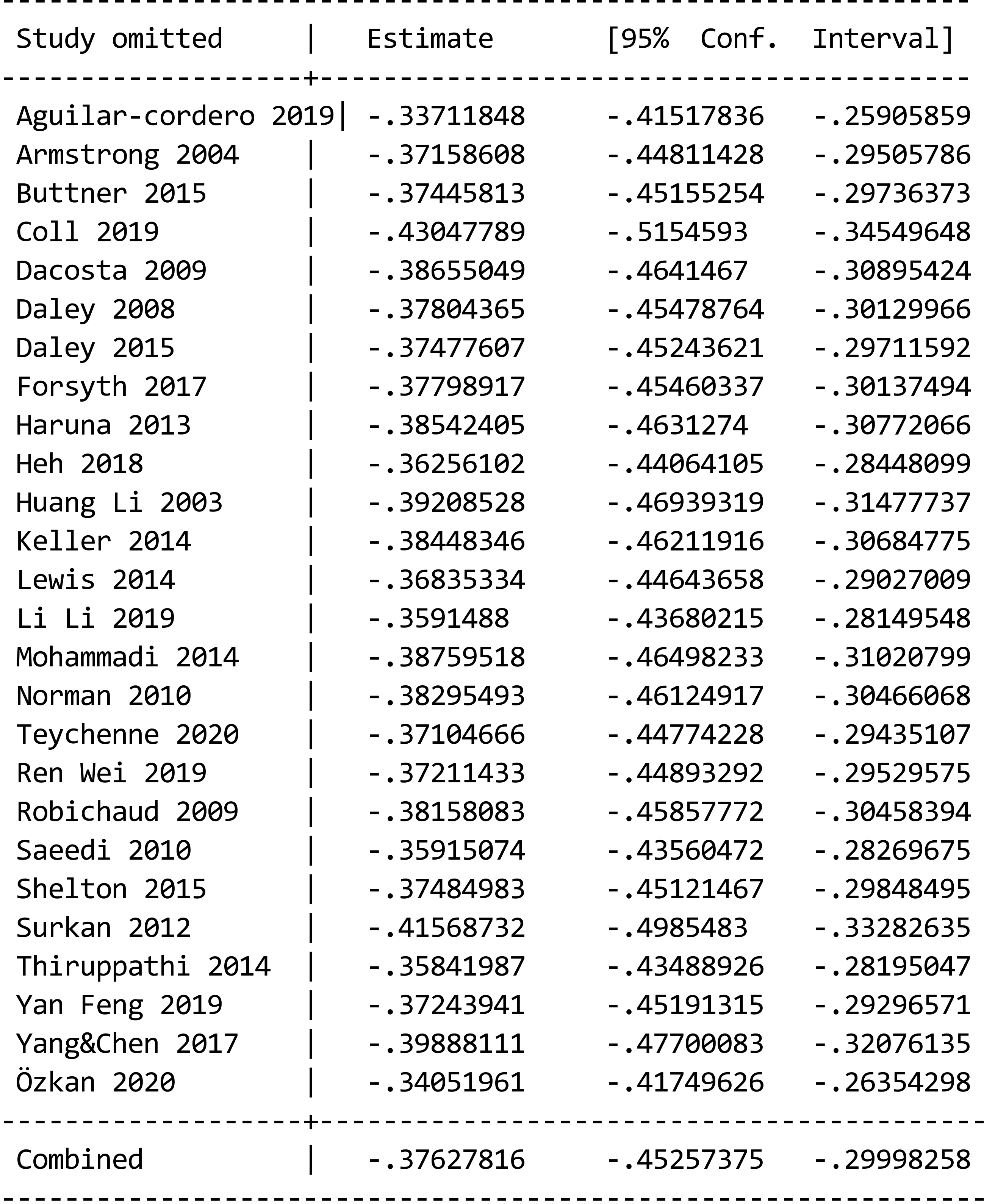
**

Supplement: S4 File — (DOCX) [file pone.0287650.s007.docx]
