# Supplementary material for: Effectiveness of aerobic exercise in the prevention and treatment of postpartum depression: Meta-analysis and network meta-analysis
Source: PLoS One. 2023 Nov 29;18(11):e0287650. doi: 10.1371/journal.pone.0287650 (PMC10686497; doi:10.1371/journal.pone.0287650)
Supplement: Attachment — Submitted filename: Response to Reviewers.docx [file pone.0287650.s008.docx]

**Dear** **Academic Editor and Reviewers,**

**Thanks for your comments of our manuscript entitled “Effectiveness of Aerobic Exercise in the Prevention and Treatment of Postpartum Depression: Meta-analysis and Network meta-analysis” (Manuscript ID PONE-D-23-04024). Those comments are all valuable and helpful for revising and improving our paper. We have discussed all comments carefully and have made conscientious revision. Below, we respond to the main comments. We also have given further detail response in the up-loaded text-revised.**

**Academic Editor (Jayonta Bhattacharjee)**

***Comment 1*.** *Please ensure that your manuscript meets PLOS ONE's style requirements, including those for file naming.*

**Response:** Thank you for your comments. We have made sure that the manuscript meets PLOS ONE's style requirements, including file naming.

***Comment 2:*** *Please state what role the funders took in the study. If the funders had no role, please state: "The funders had no role in study design, data collection and analysis, decision to publish, or preparation of the manuscript." If this statement is not correct you must amend it as needed. Please include this amended Role of Funder statement in your cover letter; we will change the online submission form on your behalf.*

**Response:** We have also included a statement about the role of the funders in the study. If the funders had no role, we will state "The funders had no role in study design, data collection and analysis, decision to publish, or preparation of the manuscript."

***Comment 3:*** *We note that you have provided funding information that is not currently declared in your Funding Statement. However, funding information should not appear in the Acknowledgments section or other areas of your manuscript. We will only publish funding information present in the Funding Statement section of the online submission form.*

**Response:** We apologize for providing funding information in the wrong section of the manuscript. We have ensured that funding information only appears in the Funding Statement section of the online submission form.

***Comment 4:*** *PLOS requires an ORCID ID for the corresponding author in Editorial Manager on papers submitted after December 6th, 2016. Please ensure that you have an ORCID ID and that it is validated in Editorial Manager. To do this, go to ‘Update my Information’ (in the upper left-hand corner of the main menu), and click on the Fetch/Validate link next to the ORCID field. This will take you to the ORCID site and allow you to create a new ID or authenticate a pre-existing ID in Editorial Manager.*

**Response:** We understand that PLOS requires an ORCID ID for the corresponding author in Editorial Manager. We have made sure that the corresponding author has an ORCID ID (0000-0003~4448-5719) and that it is validated in Editorial Manager.

***Comment 5:*** *Please review your reference list to ensure that it is complete and correct. If you have cited papers that have been retracted, please include the rationale for doing so in the manuscript text, or remove these references and replace them with relevant current references. Any changes to the reference list should be mentioned in the rebuttal letter that accompanies your revised manuscript. If you need to cite a retracted article, indicate the article’s retracted status in the References list and also include a citation and full reference for the retraction notice.*

**Response:** We have reviewed the reference list as required to ensure its completeness and accuracy, and made individual modifications in accordance with the journal's reference format requirements, such as adding the number of authors, DOIs, and web links to the references. We have not cited any retracted articles. (Page 12-16 line 398-615 of the Revised Manuscript with Track Changes).

**Reviewer 1**

***Comment 1:*** *Is the study registered? for example in prospero?*

**Response:** Thank you for your positive comments. We have been registered on *the official Prospero website*, and the registration code is CRD42023398221 ([http://www.crd.york.ac.uk/prospero/#recordDetails](http://www.crd.york.ac.uk/prospero/%23recordDetails)). The contents have been added in page 1 line 18-19 of the Revised Manuscript with Track Changes.

***Comment 2:*** *I consider that there is a lack of information in the methodological part: type of studies, language, years for the selection criteria. (much more specific)*

**Response:** We included randomized controlled trials (RCTs) that evaluated the prevention and treatment effects of aerobic exercise on postpartum depression in women, which were published between 2000 and 2023 and published in English and Chinese languages and eligible for inclusion in the meta-analysis. The contents have been added in page 3 line 90-94 of the Revised Manuscript with Track Changes.

**Reviewer 2**
***Comment 1:*** *The final section of the introduction uses literary examples to show how aerobic exercise reduces PPD depressed symptoms. There is a lot of literature, but it is not particularly connected to the study topics that were later proposed. It is advised to summarize the literature rather than simply introduce the content.*
**Response:** Thank you for your positive comments. We have formulated a comprehensive summary of the closely related references for this research topic based on the reviewer’s suggestions and have adjusted statement in page 2-3 line 66-84 of the Revised Manuscript with Track Changes.

***Comment 2:*** *In the inclusion criteria, (1) the age of the subjects is unclear; (2) the postpartum depression diagnostic criteria are ambiguous; (3) it is* *inappropriate to include depression and depressive symptoms in the study at the same time because their outcome indicators differ. It is therefore recommended to investigate them separately；(4) There was no subject designation for depression or depressive symptoms in the Table 1.*

**Response:** (1) We have revised the inclusion criteria and recruited subjects who were pregnant women of an appropriate age, usually ranging from 20 to 36 years. (Page 3 line 100-102 of the Revised Manuscript with Track Changes).

(2) Participants in the study are screened for postpartum depression (PPD) using the EPDS questionnaire, and their depressive symptoms are evaluated based on the questionnaire scores. (Page 3 line 105-106 of the Revised Manuscript with Track Changes)

(3) We apologize for including both depression and depressive symptoms in the study, which was inappropriate. Although there is some correlation between major depression and depressive symptoms as an outcome measure, they are not identical concepts. This study is limited by the fact that both were included, which may be an important factor contributing to the high heterogeneity in the meta-analysis. Therefore, we are deeply grateful for the suggestions made by the reviewers and have made the following adjustments: After carefully reviewing the 26 studies included in our analysis, we found that none of them differentiated between participants diagnosed with postpartum depression (PPD) and those exhibiting PPD symptoms (EPDS baseline) when selecting the experimental population. We have therefore redefined our study population with a more rigorous terminology, describing them as “pregnant or postpartum women with severe or mild PPD symptoms”. In addition, we will use the EPDS scoring system as the outcome measure. (Page 3 line 100-102 of the Revised Manuscript with Track Changes)

(4) The randomized controlled trials (RCTs) included in this study recruited participants based on the severity of their postpartum depression (PPD) symptoms, as indicated by their EPDS baseline. Additional details about the EPDS baseline of the subjects included in Table 1 have been provided. (Page 8 of the Figure file; Table 1 of the Revised Figure)

***Comment 3:*** *What are the classification criteria for the swimming, dance, cycling/walking/running, and yoga groups, whether by intensity, skill requirement, or other classification criteria? Please elaborate in the research. Furthermore, there are many studies on yoga in the included literature, which is very different from aerobic exercise, in the included literature.*

**Response:** The practice of categorizing aerobic exercises based on different skill requirements includes swimming, dancing, cycling/walking/running, and yoga. Pregnant women commonly opt for yoga as an aerobic exercise; however, network meta-analysis results demonstrate that its intervention is less effective in comparison to other aerobic exercise routines such as dance, swimming, cycling, walking, and running. It is imperative to acknowledge that the effectiveness of aerobic exercise interventions for PPD is not exclusively reliant on exercise types, amounts, or modes. As such, the intervention effect of yoga is subject to the influence of these comprehensive factors, with the precise reasons necessitating further study (Page 9-10 line 318-331 of the Revised Manuscript with Track Changes).

***Comment 4:*** *How should the heterogeneity of prenatal exercise be explained after subgroup analysis in the discussion?*
**Response:** The heterogeneity of the prenatal exercise group has been explained and supplemented in the discussion section of the article, with the specific reasons mainly including the following two points: (1) Compared with postpartum, pregnant women had larger differences in the severity of depressive symptoms during pregnancy, resulting in larger fluctuations in EPDS scores of the included studies. (2) Through network meta-analysis, it was found that the exercise intervention schemes of the prenatal intervention group were quite different, and the exercise types, intensity-duration combinations and exercise frequencies involved would affect the effect of aerobic exercise on postpartum depressive symptoms. (Page 11 line 363-379 of the Revised Manuscript with Track Changes).

***Comment 5:*** *Please incorporate the physiological mechanism for aerobic exercise's beneficial impact on PPD depressed symptoms.*
**Response:** We have provided a supplement in the discussion section on the physiological mechanisms of the beneficial effect of aerobic exercise on PPD depressive symptoms. (Page 10-11 line 350-362 of the Revised Manuscript with Track Changes)

**Reviewer 3**
***Comment 1:*** *However, the authors did not mention the potential side effect/risk of aerobic exercise for this group participants.*

**Response:** Thank you for your positive comments. We have supplemented this in the discussion and conclusion sections: Potential adverse effects/risks that might be experienced by participants during aerobic exercise can be avoided through the control and supervision of exercise intensity. This study explores the effects of aerobic exercise interventions on PPD while ensuring that such exercise is beneficial to both mothers and fetuses, as well as minimizing potential adverse effects and risks. (Page 10 line 333-334 and Page 10-11 line 358-360 and Page 11 line 388-393 of the Revised Manuscript with Track Changes).
