# Supplementary material for: Effectiveness of aerobic exercise in the prevention and treatment of postpartum depression: Meta-analysis and network meta-analysis
Source: PLoS One. 2023 Nov 29;18(11):e0287650. doi: 10.1371/journal.pone.0287650 (PMC10686497; doi:10.1371/journal.pone.0287650)
Supplement: Attachment — Submitted filename: Response to Reviewers.docx [file pone.0287650.s009.docx]

**Dear** **Academic Editor,**

**Thanks for your comments of our manuscript entitled “Effectiveness of Aerobic Exercise in the Prevention and Treatment of Postpartum Depression: Meta-analysis and Network meta-analysis” (Manuscript ID PONE-D-23-04024R1). Those comments are all valuable and helpful for revising and improving our paper. We have discussed all comments carefully and have made conscientious revision. Below, we respond to the main comments. We also have given further detail response in the up-loaded text-revised.**

**Academic Editor (Jayonta Bhattacharjee)**

***Comment 1*.** *Please review your reference list to ensure that it is complete and correct.*

**Response:** Thank you for your comments. We have reviewed the reference list as required to ensure its completeness and accuracy, and made individual modifications in accordance with the journal’s reference format requirements, such as adding the number of authors, DOIs, and web links to the references. We have not cited any retracted articles.

***Comment 2:*** *I have concerns about the following minor issues. I therefore request that you revise the text to fix the grammatical errors and improve the overall readability of the text. Authors also need to carefully read the manuscript again for the accuracy of references. Here are some minor corrections.
Line 21, Results: The line should start with word ‘Twenty-Six’ rather than 26.
Line 139-140: There is a repetition of a sentence.
Line 159-160: It seems ‘And’ in the staring of the sentence is not necessary.
Line 162: The line should start with word ‘Twenty-Six’ rather than 26.
Line 163: It should be from 2003. Reference number 23 seems from the year 2003. Please carefully check all the references.*

**Response:** We deeply apologize for the lack of rigor in the editing of the submitted paper and have made careful revisions based on all the suggestions made by the academic editor (Jayonta Bhattacharjee). (Line 21, Line 134-135, Line 152-153, Line 156 and Line 158 of the Revised Manuscript with Track Changes).
