# Supplementary material for: Effectiveness of aerobic exercise in the prevention and treatment of postpartum depression: Meta-analysis and network meta-analysis
Source: PLoS One. 2023 Nov 29;18(11):e0287650. doi: 10.1371/journal.pone.0287650 (PMC10686497; doi:10.1371/journal.pone.0287650)
Supplement: Attachment — Submitted filename: Response to Reviewers.docx [file pone.0287650.s010.docx]

**Dear** **Academic Editor,**

**Thanks for your comments of our manuscript entitled “Effectiveness of Aerobic Exercise in the Prevention and Treatment of Postpartum Depression: Meta-analysis and Network meta-analysis” (Manuscript ID PONE-D-23-04024R2). Those comments are all valuable and helpful for revising and improving our paper. We have discussed all comments carefully and have made conscientious revision. Below, we respond to the main comments. We also have given further detail response in the up-loaded text-revised.**

**Academic Editor (Jayonta Bhattacharjee)**

***Comment 1*.** *Please review your reference list to ensure that it is complete and correct.*

**Response:** Thank you for your comments. We have reviewed the reference list as required to ensure its completeness and accuracy, and made individual modifications in accordance with the journal’s reference format requirements, such as adding the number of authors, DOIs, and web links to the references (Page 13 line 473-476 and Page 14 line 511-516 and Page 15 line 561-564 of the Revised Manuscript with Track Changes). We have not cited any retracted articles.

**Reviewer 4**

***Comment 1:*** *Although some subgroup interventions did not yield significant comparisons, taken together, the results demonstrated that the aerobic exercise intervention is an effective tool for the prevention and treatment of PPD. Also, the effect of aerobic exercise on PPD stems from the combined effect of multiple variables within the exercise prescription. The investigators also examined multiple possibilities which result in the SUCRA scores and plots for a network meta-analysis examination of the results. The paper is well presented and the results appear to follow from all the elements performed given the systematic review and meta-analysis. Most of the edits required by the authors have been incorporated into the manuscript. However, there do remain some minor edits to be addressed. For example, on page 5, line 168 the word, ‘ridk’ should be ‘risk’.*

**Response:** We sincerely appreciate your review and valuable feedback on our manuscript. We have carefully considered your comments and have incorporated most of the edits into the revised version of the paper. We deeply apologize for the oversight regarding the word ‘rick’ on page 5, line 168. We have made the necessary correction, replacing it with ‘risk’ as suggested. We also modified some punctuation mark in the article.

We are delighted to hear that you found our study’s overall results to demonstrate the efficacy of aerobic exercise intervention as an effective tool for the prevention and treatment of postpartum depression (PPD). We acknowledge that while some subgroup interventions did not yield significant comparisons, when considered collectively, the results provide strong evidence supporting the effectiveness of aerobic exercise intervention for PPD. Furthermore, we agree with your assessment that the effect of aerobic exercise on PPD is influenced by multiple variables within the exercise prescription. We also appreciate your recognition of the comprehensive approach we employed, examining various possibilities, which led to the SUCRA scores and plots utilized in the network meta-analysis of the results.

Once again, we extend our heartfelt thanks for your time and valuable input.
