# Supplementary material for: Effectiveness of aerobic exercise in the prevention and treatment of postpartum depression: Meta-analysis and network meta-analysis
Source: PLoS One. 2023 Nov 29;18(11):e0287650. doi: 10.1371/journal.pone.0287650 (PMC10686497; doi:10.1371/journal.pone.0287650)
Supplement: Attachment — Submitted filename: Response to Reviewer.docx [file pone.0287650.s011.docx]

Dear Reviewer,

We would like to express our gratitude for your valuable comments on our manuscript titled "Effectiveness of Aerobic Exercise in the Prevention and Treatment of Postpartum Depression: Meta-analysis and Network meta-analysis" (Revision required [PONE-D-23-04024R3]- [EMID: a8940538520f27ef]). These comments have greatly contributed to the revision and improvement of our manuscript. We have diligently considered all the comments and made substantial revisions accordingly. In the following sections, we address the major comments. Additionally, we have provided more detailed responses in the uploaded revised version of the manuscript.

***Abstract***

*1. The background section seems to indicate this meta-analysis is focusing on exercise during pregnancy but some studies included also examine exercise postpartum so this should be clarified.*

**Response**: In light of this matter, we have made revisions to the background section. The exercise interventions employed in this study serve two purposes: one is to prevent postpartum depression, while the other focuses on treating postpartum depression, without specifying a specific exercise period. It's worth noting that this study encompasses both pregnant women and postpartum women as subjects. As a result, the timing of exercise for this population could occur during pregnancy, postpartum, or even both. Consequently, specifying a particular exercise period for pregnant and postpartum women could lead to confusion. Taking into account the aforementioned issues, we have made appropriate adjustments. Therefore, the expression here was indeed incorrect. It has now been revised to align with the overall context of the manuscript. (Page 1, line 9-17).

*2. In Figure 3, the overall effect appears to be -1.90 but is reported as -2.36 (similar error in results section).*

**Response**: The data has been revised and is consistent with the charts and figures. (Page 1, line 33)

*3. The first sentence of the results should indicate the comparison group (i.e., postpartum people in non-exercise interventions or treatment as usual).*

**Response:** We have improved the sentences in accordance with the above requests. (Page 1, line 30-31)

*4. The report of subgroup analysis results is misleading. Overall effects of each subgroup are reported instead of the comparison between subgroups. Therefore, the claim that these subgroups are “more beneficial” cannot be made as the subgroups are not significantly different from each other. If the overall effect of one subgroup is reported, the other subgroup should also be included. For example, overall effects of the individual exercise subgroup were also significant as compared to control, so there is little justification for promoting only the value of team based exercise. It would be best practice to report the test for subgroup differences instead of overall effects of the individual subgroups.*

**Response**: The manuscript has been revised to incorporate the significance results of "Test for subgroup difference" and to restate the results of subgroup analysis by considering the combined effect sizes (Subtotal 95%CL) of each subgroup. For example: “Subgroup analysis suggests that the intervention objective (prevention vs. treatment) of exercise could potentially be a source of heterogeneity in this study，as the “Test for subgroup difference” revealed the presence of significant distinctions (p=0.02＜0.05). The “Test for subgroup difference” yielded non-significant results for both the supervised vs. unsupervised subgroup comparison (p=0.55 > 0.05) and the individual vs. team subgroup comparison (p=0.78 > 0.05). Nonetheless, when assessing their effect sizes [Subtotal (95%CL)], the supervised exercise group [-1.66 (-2.48, -0.85)] exhibited a slightly better performance than the unsupervised exercise group [-1.37 (-1.86, -0.88)], while the team exercise group [-1.43 (-1.94, -0.93)] slightly outperformed the individual exercise group [-1.28 (-2.23, -0.33)].” (Page 2, line 34-42)

*5. If reporting the overall effect for supervised exercise, it seems the p value is incorrect (from the heterogeneity test).*

**Response:** The manuscript has been uniformly revised in terms of expressing heterogeneity and effect sizes. In Figure 5, a total of 4 p-values are presented, representing the heterogeneity within the supervised exercise group, the heterogeneity within the unsupervised exercise group, the overall heterogeneity across the two groups of studies, and finally, the statistical significance of the "Test for subgroup difference" regarding the differences between the two groups' outcomes. In the heterogeneity assessment of the Meta-analysis, the p-value and the meaning represented by I^2^ for the "supervised exercise" group are consistent. They both indicate whether there is statistical significance in the variability of study results within a specific subgroup, rather than indicating the significance testing between the experimental and control groups in all studies. The summarized results of the experimental and control groups for all studies are represented by Overall Effects (MD) and 95%CL, presented intuitively through a forest plot. For this subgroup, the p-value is 0.12 > 0.05 from the heterogeneity test of subgroup, and I^2^ is 37% < 50%；the overall effect for supervised exercise is presented as (Subtotal 95%CL), and corresponding p-values are not provided. Instead, the significance of intervention effects is determined by whether the 95% confidence interval (95%CL) includes 0. This indicates that the variability of results within all studies included in this subgroup is not statistically significant, and it can also be considered to have good consistency. To avoid misinterpretation, the heterogeneity across all studies is indicated using the I^2^ statistic，subgroup effect sizes are all presented as "MD 95%CL."(Page 2, line 34-40)

*6. The final sentence of the results should note that this is in comparison to other frequencies/intensities.*

**Response:** The requested changes have been made. (Page 2, line 46-53)

*7. As noted for the results section, the conclusion regarding subgroups is misleading given lack of significant difference.*

**Response:** The subgroup analysis results have been reinterpreted by incorporating the significance results of the "Test for subgroup difference" and the combined effect sizes (Subtotal 95%CL) of each subgroup. (Page 2, line 64-66)

*8. The final sentence suggests that the benefits are just during pregnancy—is this the case for all the studies included in the comparisons? Or were some postpartum?*

**Response:** In fact, this study focuses on both pregnant women and postpartum women as subjects. Therefore, the timing of exercise for pregnant and postpartum women could be during pregnancy, postpartum, or even both. Consequently, we should not specify the timing of exercise for this population to avoid confusion. We have made new adjustments considering the issues mentioned earlier. (Page 2, line 61-62)

***Introduction***

*1. Line 46: The reported prevalence range is very large, which decreases the meaningfulness of the statement. Is there a prevalence that may be most accurate? Or it would be helpful to explain this wide range.*

**Response**: The optimal revisions have been made according to the reviewer's suggestions. (Page 3, line 77-80)

*2. The final two sentences of the introduction would be better suited for the discussion. Instead, it would be useful to include a priori hypotheses.*

**Response:** The most appropriate modifications have been made as per the reviewer's suggestions. (Page 4, line 110-117)

*Methods*

*1. What is the rationale for the timeframe for the publications being after 2000?*

**Response:** We realized that setting the search start date to the year 2000 could potentially lead to the omission of some studies. Therefore, we conducted a search and update of literature from the inception of the databases, but did not find any studies that met the inclusion criteria before the year 2000. As a result, we are now changing the literature search time frame to the inception of the databases. (Page 4, line 126-130)

*2. What is the rationale for the need to include both “postpartum depression” AND “maternal depression” in title or abstract. I would imagine this might exclude some studies that had one or the other terms.*

**Response:** After examining the detailed search strategy in the supplementary materials, we have identified writing oversights in the manuscript regarding the information in this section. Here, in fact, it should be "OR." The error has already been corrected in the manuscript. (Page 4, line 131)

*3. What is the rationale for the age range up to 36 years old?*

**Response:** Originally, the ideal age range for the inclusion criteria of the study participants was intended to be within the age range of eligible pregnant and postpartum women. However, due to the limited number of literatures meeting these criteria, the age range was eventually narrowed down to participants aged 18 and above. Unfortunately, during the writing process, the study editor did not promptly update the inclusion criteria, resulting in an error due to outdated information. The inclusion criteria for participant age have now been corrected to be 18 years and above (adult females). (Page 4, line 137)

*4. Were moderate depression symptoms included? Only mild or severe are indicated in inclusion criteria.*

**Response:** Due to the same reason as in Question 3, the intended meaning of this English sentence does not accurately convey the author's final intention. The entire sentence has been revised to: “The participants are normal pregnant women or postpartum depression patients who are adults (≥18 years).” (Page 4, line 136-137)

*5. The EPDS is referred to differently in lines 101 (P=postpartum) vs. 120/122 (postnatal).*

Response: The use of this term has been standardized throughout “postnatal”. (Page 5, line 142)

*6. Statistical analysis/figures: It is unclear to me what the mean/SD for the experimental group represents vs. mean/SD for control group. Are these mean scores at post-exercise intervention? Mean changes from pre to post intervention? In the statistical analysis section, it reports “comparing scores before and after the aerobic exercise intervention.” It should be clarified if the means being compared are mean changes from pre to post or something else.*

**Response:** In reality, the term "outcome data" here refers to the scores on the Edinburgh Postnatal Depression Scale for the experimental and control groups after exercise interventions, represented by means and standard deviations. Based on this data, we conducted Meta-analysis to calculate the effect sizes (MD) and 95% confidence intervals for the experimental and control groups in each study. By aggregating all these results, we can determine whether exercise intervention is effective in preventing and treating postpartum depression. This determination relies on whether there is a significant difference in the outcomes between the experimental and control groups in the Meta-analysis. The erroneous sentence concerning the expression of outcome data for the experimental and control groups after exercise intervention has been corrected. To enhance understanding, the explanation and clarification of the data analysis have been reinforced. (Page 6, line 210-217)

***Results***

*1. As noted previously, the overall effect (line 171) appears to be misreported if the numbers in Figure 3 are correct.*

**Response:** The numerical values in the text have been corrected according to the data in the figures. (Page 9-10, line 260-263)

*2. Section 3.3.1 should note the comparison group for the effect*

**Response:** The requested supplementation has been completed. (Page 9-10, line 263-263)

*3. Please adjust to be a complete sentence: The combined effect size for the aerobic exercise effect on postpartum depression intervention was (26 RCTs; MD= -2.36, 95% CL: -2.58 to -2.15; p<0.00001).*

**Response:** The requested improvements have been made as per the requirements. (Page 9-10, line 260-261)

*4. For all subgroup analyses and network analyses, please indicate what “other intervening variables” mean.*

Response: “other intervening variables” This indicates that factors other than the grouping variable need to be controlled to account for potential influences on the outcomes. This can be achieved through methods such as random allocation and adjustments through matching. For instance, in the context of the grouping variable comparing supervised vs. unsupervised, it is important to rigorously control other potential influencing factors between the two groups, such as the intervention purpose (prevention vs. treatment), exercise volume (type, intensity, duration, frequency), and exercise organization (team vs. individual). Random allocation ensures that factors other than supervision itself are balanced between the supervised and unsupervised groups, with strict differentiation solely based on the "supervision" factor. This portion of content has been added to the Grouping Methods section as requested. (Page 10, line 272-273,286-287,298-299,312-313,336-337,355-356,)

*5. As noted previously, subgroup results are misleading as reported. The tests for subgroup differences indicate no significant differences in team vs. individual exercise (p=.78) or supervised vs. unsupervised exercise (p=.55). There is, however, a significant difference in prenatal vs. postnatal exercise (p=.02). To be most transparent, these subgroup differences should be reported in the results if authors want to say “more beneficial” or “less beneficial” as those are the appropriate tests for those conclusions. Authors may also choose to report the overall effect for each subgroup and relative direction of the effect but should not make the claim that one subgroup is “more beneficial” if the subgroup analysis is not significant. This type of conclusion is especially misleading for team vs. individual exercise in which both subgroups have significant overall effects as compared to controls, which ultimately means either could be recommended as a good source of exercise to reduce postpartum symptoms. This can be similarly said of supervised vs. unsupervised exercise as well.*

**Response:** The subgroup analysis section has been accurately summarized based on the comprehensive results of the "Test for subgroup difference" and "Subtotal 95%CL." For example, Subgroup analysis suggests that the intervention objective (prevention vs. treatment) of exercise could potentially be a source of heterogeneity in this study，as the “Test for subgroup difference” revealed the presence of significant distinctions (p=0.02＜0.05). The “Test for subgroup difference” yielded non-significant results for both the supervised vs. unsupervised subgroup comparison (p=0.55 > 0.05) and the individual vs. team subgroup comparison (p=0.78 > 0.05). Nonetheless, when assessing their effect sizes [Subtotal (95%CL)], the supervised exercise group [-1.66 (-2.48, -0.85)] exhibited a slightly better performance than the unsupervised exercise group [-1.37 (-1.86, -0.88)], while the team exercise group [-1.43 (-1.94, -0.93)] slightly outperformed the individual exercise group [-1.28 (-2.23, -0.33)]. (Page 10, line 275-277,287-293,201-306,)

*6. Incorrect p values are reported for* *supervised and unsupervised exercise overall effects.*

**Response:** Here, we should focus on reporting whether the differences between subgroups are statistically significant, specifically the p-values of “Test for subgroup difference”. The error in reporting at this point has been corrected. The overall effect for supervised exercise is presented as (Subtotal 95%CL), and corresponding p-values are not provided. Instead, the significance of intervention effects is determined by whether the 95% confidence interval (95%CL) includes 0. (Page 10, line 287-293)

*7. It is unclear why cycling, walking, and running are grouped together. Walking and running/cycling would likely have different intensities and ultimately different effects (especially since intensity did yield differential results).*

**Response:** The methodology section (Added Section 2.4. Grouping criteria) of the manuscript has been explained：“An elucidation is warranted for categorizing cycling/walking/running as a singular exercise type. This decision is attributed to the relatively infrequent occurrence of these activities in isolation. Exercise guidelines often combine cycling/walking or running (jogging) /walking within training plans. Additionally, by considering the weekly exercise volume involving all these aerobic exercises, the groups with similar weekly exercise volume are classified under yoga, dance, and swimming categories, while the less frequent exercise types are grouped together.” (Page 6, line 191-197)

*8. For exercise frequency, was this defined as the actual frequency with which individuals completed exercise or the prescribed frequency? If the latter, though this frequency may be prescribed, it is likely that many individuals within the study did not meet this frequency. This should be clarified and noted within the discussion/limitations if the latter. Similar note for exercise intensity in terms of actual vs. prescribed.*

**Response:** The discussed exercise volume (exercise frequency, type, duration, intensity) in this study refers to the prescribed exercise volume in the exercise program, rather than the actual completed exercise volume by the participants. This clarification has been added to the discussion section of the manuscript. This study lacks relevant data on exercise adherence among the experimental participants, thus preventing us from determining the actual completion status of the intervention protocols. Our current discussion solely focuses on the prescribed exercise volume within the exercise plans, rather than the actual exercise volume accomplished by the participants. Whether it’s the prescribed exercise volume or the actual achieved volume, both ultimately need to be tailored according to the physical capabilities and conditions of the pregnant and postpartum women. The planned exercise volume serves as a reference for safe exercise, and holds significance and value. Therefore, engaging in exercise 3 to 4 times per week, along with moderate exercise intensity and a duration of 35 to 45 minutes, represents an optimal prescribed exercise volume, offering guidance for exercise prescription. However, it may not necessarily reflect the actual exercise volume achieved by all participants. (Page 16, line 479-489)

*9. For ease of understanding, exercise intensities should also be described in terms of low, moderate, high.*

**Response:** The wording related to exercise intensity has been changed as requested: “low exercise intensity: 40% HRR; moderate exercise intensity: 50%~ 60% HRR; high exercise intensity: 65% ~ 74% HRR” (Page 13, line 372)

*10. How were the exercise intensity/duration groupings made?*

***R*esponse:** The methodology section (Added Section 2.4. Grouping criteria) has been supplemented with the relevant explanations: “Exercise volume are arranged in three levels, descending from high to low volume. Based on exercise frequency, they are stratified into three categories: 2~3 times per week, 3~4 times per week, and 5-6 times per week. Notably, upon scrutinizing the planned exercise intensities and durations across all the included studies, a pattern emerges where higher exercise intensities are often paired with shorter exercise durations. Aligning similar patterns of exercise intensity and duration culminates in three categories: high intensity (50~60 minutes), moderate intensity (35~45 minutes), and low intensity (20~30 minutes).” (Page 6, line 202-206)

*11. In section 3.6, p for publication bias is listed as .23 but Table 6 says .32.*

*Response: The numerical values have been updated and are consistent with Table 5* (Page 13, line 386)

*.*

***Discussion***

*1. As noted previously, given lack of significant differences between subgroups, team exercise and supervised exercise cannot be described as increasing effectiveness of the interventions. If the discussion is to theorize why team exercise had a better overall effect than control, this is fine, but should be clearly stated. Additionally, since individual exercise also had better effect than control, it should equally be included in the discussion for possible mechanisms. This is similar re: supervised vs. unsupervised exercise given significant overall effects of the individual subgroups and lack of significant differences between the subgroups.*

**Response:** The results of the subgroup analysis have been comprehensively supplemented and improved. Consequently, the discussion section has been completely rewritten to reflect the new information. The main points covered include the reasons for the lack of significant differences in subgroups, the implications of the network meta-analysis regarding prescribed exercise volume, and the limitations of this study. (Page 14, line 399-410)

*2. In general, the discussion is far too prescriptive.*

**Response:** Regarding the main concerns of this study, the discussion section has been reanalyzed comprehensively and in-depth. (Page 14-16, line 391-498)

*3. Physiological mechanisms are proposed and include psychological components. If including psychological components (e.g., bullet 3), the vast literature on mood benefits should also be noted.*

**Response:** Due to the realization that the current focus of the discussion is not suitable for including discussions about the physiological mechanisms, the section related to the mechanisms of exercise intervention for postpartum depression, along with relevant references, has been ultimately removed. If the reviewing experts find this part necessary, we can consider adding it back based on their suggestions. However, we have noted the vast literature on mood benefits and have also added relevant references. (Page 15, line 420-433)

*4. Limitations are minimally mentioned and the discussion of limitations should be expanded.*

**Response:** The limitations of this study have been analyzed throughout the entire discussion section. (Page 15-16, line 449-463,490-498)

***Conclusions***

*1. Similar notes to previously stated re: interpretations of subgroup analyses and being far too prescriptive (i.e., not balancing potential limitations of the data).*

**Response:** Regarding this issue, the discussion section has been comprehensively and thoroughly analyzed. (Page 19, line 591-592)

*2. Expansion of future directions would be beneficial in discussion (and possibly conclusions).*

**Response:** Based on the suggestions provided, the discussion section has undergone a comprehensive and in-depth analysis. (Page 19, line 593-607)

***Tables***

*1. Can Table 1 be put in landscape? It is very hard to follow as written.*

**Response:** The positioning of figures and tables can be adjusted as per the preferences of the journal's production department.

*2. For the EPDS, what does the mean represent? Is this the mean at the end of the intervention? Or mean change? Mean change would be more ideal as this would account for baseline levels of depression.*

**Response:** This is the mean at the end of the intervention. The term "outcome data" here refers to the scores on the Edinburgh Postnatal Depression Scale for the experimental and control groups after exercise interventions, represented by means and standard deviations. We conducted Meta-analysis based on this data, which allowed us to calculate the effect sizes and 95% confidence intervals for the experimental and control groups in each study. These calculations help determine the intervention effect resulting from exercise intervention, comparing the experimental and control groups. (Page 9, line 249-251)

*3. What is the rationale for categorizing exercise classes as “team”? Is this better defined as individual vs. group? It may be helpful to define these terms in the methods.*

**Response:** All grouping criteria have been described in the methodology section (Added Section 2.4. Grouping criteria) of this study. (Page 5, line 178-183)

*4. The inclusion criteria note that age range should be 20-36; however, it appears mean age for Yan is 36.6?*

**Response:** Regarding this issue, adjustments have already been made to the inclusion criteria. (Page 5, line 168)

We firmly believe that these revisions further enhance the quality and readability of the manuscript. Once again, we extend our heartfelt thanks for your time and valuable input. Should you have any further questions or requests, please do not hesitate to contact us. We also modified some punctuation mark in the manuscript.

Best regards,

Hao Xu, Renyi Liu
