# Supplementary material for: Effectiveness of aerobic exercise in the prevention and treatment of postpartum depression: Meta-analysis and network meta-analysis
Source: PLoS One. 2023 Nov 29;18(11):e0287650. doi: 10.1371/journal.pone.0287650 (PMC10686497; doi:10.1371/journal.pone.0287650)
Supplement: Attachment — Submitted filename: Response to Reviewers.docx [file pone.0287650.s012.docx]

**Dear** **Academic Editor and Reviewer,**

**Thanks for your comments of our manuscript entitled “Effectiveness of Aerobic Exercise in the Prevention and Treatment of Postpartum Depression: Meta-analysis and Network meta-analysis” [PONE-D-23-04024R4] - [EMID: aa83befb7a1a3e58]. Those comments are all valuable and helpful for revising and improving our paper. We have discussed all comments carefully and have made conscientious revision. Below, we respond to the main comments. We also have given further detail response in the up-loaded text-revised.**

**Academic Editor (Jayonta Bhattacharjee)**

***Comment 1*.** *Please review your reference list to ensure that it is complete and correct. If you have cited papers that have been retracted, please include the rationale for doing so in the manuscript text, or remove these references and replace them with relevant current references. Any changes to the reference list should be mentioned in the rebuttal letter that accompanies your revised manuscript. If you need to cite a retracted article, indicate the article’s retracted status in the References list and also include a citation and full reference for the retraction notice.*

**Response:** We have carefully reviewed the reference list for our manuscript and have taken the necessary steps to ensure that it is complete and accurate. We have confirmed that none of the cited papers have been retracted, and therefore, there is no need to include a rationale for their inclusion in the manuscript text. Due to the expanded discussion section, two additional references have been added to the reference list. (Line 664-671 of the revised manuscript with track changes) We have not cited any retracted articles, so there is no need to indicate the retracted status in the References list or include retraction notices. We appreciate the thorough review process and are committed to maintaining the integrity of our references in accordance with the journal's guidelines. Thank you for your attention to this matter.

**Reviewer 5**

Thank you very much for your positive feedback on the revised manuscript and for your valuable suggestions. We will work to improve the manuscript according to your recommendations. Once again, we appreciate your patience and diligent work. Thank you for your assistance and support.

***Results***

***Comment*** ***1****. For the network meta-analysis, section 3.4.2 a)—please label as “**prescribed frequency” to ensure understanding this was prescribed vs. actual. It is appreciated this has been added to the discussion.*

**Response:** The modifications have been made as requested. (Line 314-333 of the revised manuscript with track changes)

***Comment 2.*** *Similarly, please label “**prescribed intensity-duration.”*

**Response:** The modifications have been made as requested. (Line 335-356 of the revised manuscript with track changes)

***Comment*** ***3.*** *You don’t necessarily have to repeat this phrase with additions to the methods data analytic section: “Under the premise of ensuring the random allocation of other variable factors apart from the…”*

**Response:** The modifications have been made as requested. (Line 290-291,316-317,337-338 of the revised manuscript with track changes)

***Discussion***

***Comment*** ***1.*** *I find the significant subgroup result of exercise performing better in prevention than postpartum to be quite interesting. If possible, it would enrich the discussion to include some thoughts as to why this might be important in terms of timing.*

**Response:** The discussion on terms of timing has been added as requested. “Subgroup analysis suggests that the intervention objective (prevention vs. treatment) of exercise could potentially be a source of heterogeneity in this study (p=0.02＜0.05), indicating that the preventive effects of aerobic exercise are superior to the therapeutic effects. It is well known that engaging in appropriate aerobic exercise during pregnancy not only promotes pelvic mobility and increases birth canal space to alleviate maternal labor pain but also helps prevent pregnancy complications. There is evidence to suggest that postpartum depression (PPD) doesn’t exclusively occur after childbirth, as population-based studies indicate a similar 12% occurrence rate of depression during pregnancy. This suggests that PPD symptoms may originate during pregnancy in certain cases [43]. Furthermore, studies indicate that the antidepressant effects of exercise can persist for a period after exercise cessation [44]. Therefore, engaging in aerobic exercise during pregnancy may have a greater impact on preventing PPD compared to exercise as a treatment postpartum.” (Line 386-400 of the revised manuscript with track changes)

***Comment*** ***2.*** *Line: 426-427: “Maternal” should say “**mothers” And maternal could be quickly assisted by other peers in the event of an emergencies such as falls or other discomfort to ensure the safety of the exercise [46].*

**Response:** The modifications have been made as requested. (Line 415 of the revised manuscript with track changes)

**We firmly believe that these revisions further enhance the quality and readability of the manuscript. Once again, we extend our heartfelt thanks for your time and valuable input. Should you have any further questions or requests, please do not hesitate to contact us. We also modified some punctuation mark in the manuscript.**

**Best regards,**

**Hao Xu, Renyi Liu**
